# Deep Eutectic Solvents for Starch Treatment

**DOI:** 10.3390/polym14020220

**Published:** 2022-01-06

**Authors:** Dorota Skowrońska, Katarzyna Wilpiszewska

**Affiliations:** Department of Organic Chemical Technology and Polymeric Materials, Faculty of Chemical Technology and Engineering, West Pomeranian University of Technology in Szczecin, ul. Pułaskiego 10, 70-322 Szczecin, Poland; kwilpi@zut.edu.pl

**Keywords:** starch, deep eutectic solvents, starch dissolution, starch plasticization

## Abstract

In this review, the application of deep eutectic solvents (DESs) as starch solvents, plasticizers and for other treatment has been described. Starch, as one of the most abundant biopolymers, is considered for forming new biodegradable materials. This new approach, referring to applying deep eutectic solvents for dissolving starch, its plasticization and other modifications, was presented. A DES could be a good alternative for common starch plasticizers (e.g., glycerol, urea) as well as recently considered ionic liquids. The high variety of DES component combinations makes it possible to obtain materials with the properties specific for given applications.

## 1. Introduction

The growing problem of environmental pollution with plastic wastes has become a motivation for research teams to look for more “green materials”, i.e., biodegradable and from renewable resources. Starch is an inexpensive and abundant biopolymer. In its native form, starch occurs in the form of semicrystalline granules, insoluble in cold water. It consists of two types of macromolecules—linear amylose and branched amylopectin. Due to the presence of inter- and intra-hydrogen bonds between polymeric chains, the degradation temperature of starch is lower than its melting temperature. The hydrogen bonds network reduces the mobility of the macromolecules. The energy necessary to increase the mobility of the chains is higher than the energy that provides polymer degradation [1]. As a consequence, starch cannot be processed by common plastic processing methods, e.g., by extrusion, injection, or compression moulding. In the presence of the substance that is able to form hydrogen bonds with polysaccharide chains (plasticizer) at an elevated temperature and shear stress, the granular structure of starch is disrupted, and thermoplastic starch (TPS) could be obtained. For many years, research on starch plasticizers, such as urea [2], glycerol and other polyols [3], sugars [4], and formamide [5], were performed. However, the mechanical properties of the obtained materials depended on the moisture content; moreover, they exhibited a tendency to retrogradation (recrystallization), and some plasticizers were toxic [6]. Recently, using a new class of “green” compounds, i.e., deep eutectic solvents (DESs), has been considered for starch treatment, e.g., for its dissolution or plasticization.

**Figure 1:**
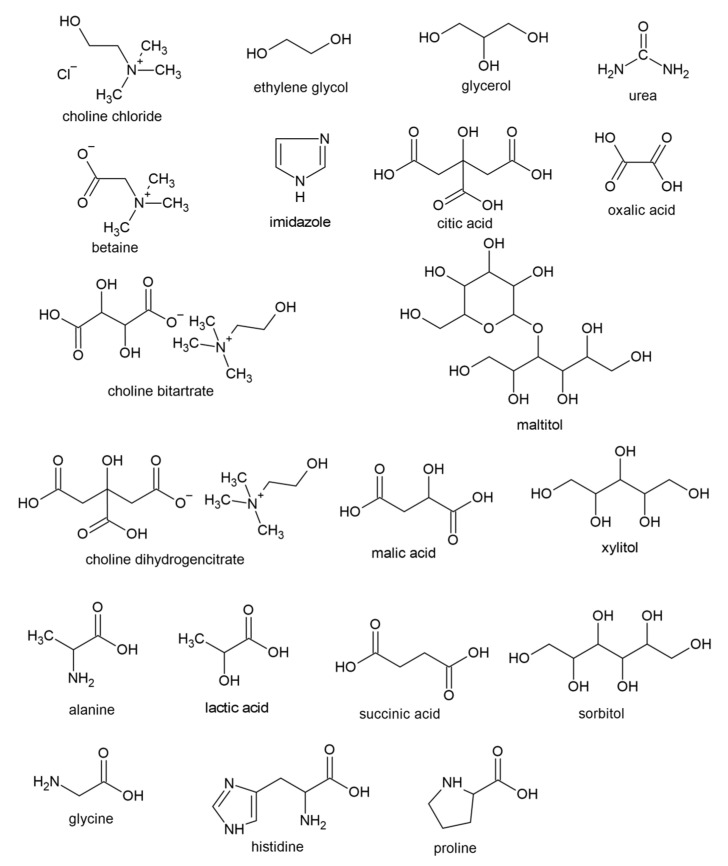
The most popular compounds of deep eutectic solvents used for starch treatment.

Deep eutectic solvents are the mixtures of so-called hydrogen bonds acceptors (HBAs) and hydrogen bonds donors (HBDs). DESs exhibit much lower melting temperatures than their components separately (in a strictly defined molar ratio of components). The difference between eutectic and deep eutectic mixtures is not defined precisely. DESs show a “marked deviation from “ideal-solution” behaviour” [7], but the mechanisms behind this are still unexplored. They share many similarities with ionic liquids, and some researchers acknowledge them as a subclass of ionic liquids. Generally, they show high thermal stability, nonflammability, low volatility, and vapor pressure. Unlike ionic liquids, they are mostly biodegradable, non-toxic, easy to prepare, and relatively inexpensive [8]. The first report on DESs was presented in 2003 [9]. Abbott et al. [9] reported a substantial decrease in the melting temperature of the eutectic mixture based on choline chloride and urea (molar ratio 1:2), called reline. Since then, an increasing interest in eutectic solvents has been observed. The deep eutectic solvents are divided into the following five classes: I—quaternary ammonium salts with metal chlorides; II—quaternary ammonium salts with metal chloride hydrates; III—quaternary ammonium salts with HBDs (e.g., polyols, amines, carboxylic acids); IV—metal chloride hydrates with HBDs; V—non-ionic mixtures of HBDs and HBAs. However, as deep eutectic solvents are a fairly new class of solvents, the terminology is still evolving. Choi et al. [10] introduced the term “natural deep eutectic solvent” (NADES), which refers to DESs based on plant metabolites, such as choline chloride, natural carboxylic acid, sugars, and water. Francisco et al. [11] proposed a “low transition temperature mixture” (LTTM) term for the mixtures that do not exhibit the eutectic point (characteristic for DESs). Moreover, the solvents used for pharmaceutical applications are called “therapeutic deep eutectic solvents” (THDESs) [12]. In Figure 1, the popular compounds of deep eutectic solvents used for starch treatment were presented.

Research on replacing the substances considered toxic or non-environmentally friendly with the DES systems is being performed. Reports on using DESs in metal processing, purification, gas adsorption, biomass treatment as well as the reaction media or modification agents can be found [13,14].

## 2. DESs as Starch Solvents

Dissolution efficiency is often considered as the preliminary test that allowed us to evaluate the potential application of the DES system for polysaccharide treatment. In Table 1, the DES systems used as starch solvents were presented.

Biswas et al. [15] studied dissolving starch in a DES (based mostly on choline chloride and the following various HBDs: oxalic acid, urea, zinc chloride, citric acid); additionally, the dissolution temperature was noted. The goal of his study was to apply a DES as a reaction medium for starch acetylation. The lowest dissolution temperature was noted for a calcium chloride and urea mixture, i.e., 80 °C.

Zdanowicz and Spychaj [16] compared dissolving potato starch in ionic liquid (1-allyl-3-methylimidazolium chloride) to the DES based on choline chloride and urea, citric acid, or succinic acid. The potato starch was dissolved without the signs of degradation (brown colour) only in the deep eutectic solvent containing urea. 

Francisco et al. [17] studied dissolving polysaccharides (starch, lignin, and cellulose) in mixtures of carboxylic acids (lactic, malic, oxalic, and nicotinic acid), and different HBAs (proline, betaine, alanine, glycine, histidine) at various temperatures. The highest yield was obtained for a mixture of glycine and malic acid (molar ratio of 1:1) at 100 °C. 

Dai et al. [18] presented over one hundred natural deep eutectic solvents and their basic analysis. The solubility of selected polymers (including starch) was tested. The highest starch solubility, i.e., 7.55 g/mol_solvent_, was noted for the ternary mixture of choline chloride, glucose, and water (molar ratio of 5:2:5) at 100 °C. 

Zdanowicz et al. [19] tested mixtures based on imidazole. Starch (10 wt% solution) was dissolved in a mixture of choline chloride with imidazole and glycerol with imidazole (molar ratio of 3:7) at 100 °C, in contrast to carboxylic acid-based systems. 

Yiin et al. [20] observed the highest starch solubility of 2.25 wt% for a mixture of malic acid, sucrose, and water (molar ratio of 1:3:10) at 60 °C. 

Zdanowicz [21] tested urea-based systems with choline derivatives with different anions as well as a ternary mixture of choline chloride, urea, and glycerol (molar ratio of 1:1:1) as starch solvents (at 110 °C). All the tested systems exhibited at least a partial ability to dissolve starch. In another work [22], Zdanowicz studied urea-based DESs (with glycerol, sorbitol, fructose, and glucose). Potato starch dissolved only in the mixture of urea with glycerol (molar ratios of 1:1 and 1:2, respectively).

**Table 1:** Deep eutectic solvents used as starch solvents.

Starch Origin	DES System		Solubility	Test Parameters	Reference
	Components	Molar Ratio			
Corn	Choline chloride: Oxalic acid	1:2	9%, (brown solution)	100 °C	[15]
	Choline chloride: Urea	1:2	9 wt%	100 °C	[15]
	Choline chloride: Zinc chloride	1:2	5 wt%	98 °C	[15]
	Choline chloride: Citric acid	1:2	6 wt%	100 °C	[15]
	Malic acid: Monosodium glutamate: Water	3:1:1, 3:1:2, 3:1:10	0.95; 1.03; 1.11 wt%	60 °C	[20]
	Malic acid: Sucrose: Water	1:1:1, 1:1:2, 1:1:10, 1:2:10, 1:3:10	0.12; 0.25; 0.31; 0.92; 2.25 wt%	60 °C	[20]
	Calcium chloride: Urea	1:2	gelled	80 °C	[15]
n.d. *	Alanine: Lactic acid	1:9	0.26 wt%	60 °C	[17]
	Alanine: Malic acid	1:1	0.59 wt%	100 °C	[17]
	Betaine: Malic acid	1:1	0.81 wt%	100 °C	[17]
	Choline chloride: 1,2-Propanediol: Water	1:1:1	2.47 g/mol_solvent_	100 °C	[18]
	Choline chloride: Glucose: Water	5:2:5	7.55 g/mol_solvent_	100 °C	[18]
	Choline chloride: Lactic acid	1:10	0.13 wt%	60 °C	[17]
	Choline chloride: Malic acid	1:1	7.10 wt%	100 °C	[17]
	Choline chloride: Oxalic acid anhydrous	1:1	0.15 wt%	60 °C	[17]
	Choline chloride: Oxalic acid dihydrate	1:1	2.50 wt%	60 °C	[17]
	Glucose: Lactic acid: Water	1:5:3	1.67 g/mol_solvent_	100 °C	[18]
	Glycine: Malic acid	1:1	7.65 wt%	100 °C	[17]
	Histidine: Lactic acid	1:9	0.13 wt%	60 °C	[17]
	Nicotinic acid: Oxalic acid dihydrate	1:9	2.83 wt%	60 °C	[17]
	Proline: Malic acid	2:1, 3:1	0.32%, 5.90%	100 °C	[17]
	Proline: Oxalic acid anhydrous	1:1	0.15 wt%	60 °C	[17]
Potato	Choline acetate: Urea	1:2	10 wt% starch, partially dissolved	110 °C, 30 min	[21]
	Choline chloride: Citric acid	2:1	5 wt%, brown viscous liquid	120 °C, 60 min	[16]
	Choline chloride: Imidazole	3:7	10 wt%, transparent gel	100 °C, 60 min	[19]
	Choline chloride: Succinic acid	1:1	5 wt% brown viscous liquid	135 °C, 60 min,	[16]
	Choline chloride: Urea	1:2	5 wt%, colourless, viscous liquid;	118 °C, 60 min	[16]
			10 wt%, dissolved	110 °C, 30 min	[21]
	Choline chloride: Urea: Glycerol	1:1:1	10 wt%, partially swollen and gelled	110 °C, 30 min	[21]
	Choline lactate: Urea	1:2	10 wt%, foamed, after one week storage transparent gel	110 °C, 30 min,	[21]
	Glycerol: Imidazole	1:1	10 wt%, slightly turbid gel	100 °C, 60 min	[19]
		3:7	10 wt%, transparent gel	100 °C, 30 min	[19]
	Urea: Glycerol	1:1	10 wt%, started to form gel 15 min at 80 °C, destruction of granule at 85 °C	80–85 °C	[22]
		1:2	10 wt%, started to form gel after 20 min at 90 °C; destruction of granule at 110 °C	90–110 °C	[22]

* no data given.

## 3. DESs for Starch Plasticization

In the plasticization process, the granular structure of starch is disrupted in the presence of an additional substance that is able to form hydrogen bonds with polysaccharide chains, i.e., a plasticizer. The starch granules swell, and the crystalline structure is interrupted. The plasticizer type affects the properties of thermoplastic starch, including a tendency to retrogradation and sensitivity to moisture [23]. The key parameters for starch plasticization are temperature, sheer force, and nature of the plasticizer, which is why the plasticization of the same system in different methods provides different results. Sheer forces influence the penetration of the plasticizer into the polymer structure most of all. Moreover, the water content in the starch material is an important parameter. However, as some water content is required for efficient polysaccharide plasticization, its excess results in the significant deterioration of mechanical properties [24]. The efficiency of plasticization is generally evaluated indirectly, e.g., using thermal methods, where the chain mobility is observed (the shift of glass transition temperature). 

Spychaj and Zdanowicz [16] compared the plasticizing ability ionic liquids to three deep eutectic solvents based on choline chloride and different hydrogen-bond donors (urea, succinic acid, and citric acid). Plasticizing was performed by thermocompression. The best plasticizing properties (in the compression moulding test) was noted for the choline chloride–urea mixture. It was also confirmed that choline chloride can be used as the only plasticizer of humid starch.

Abbot et al. [25] studied the plasticization of starch with a DES consisting of choline chloride and urea with a molar ratio of 1:2 (reline). The different appearance of starch plasticized with and without applying pressure was reported to be transparent and opaque, respectively. It was suggested that using pressure resulted in the plasticized penetrating between the polysaccharide chains better. Moreover, the effect of applying various techniques for starch plasticization was evaluated, i.e., by extrusion and thermocompression. Extruded starch exhibited better mechanical properties—a higher Young’s modulus, and ultimate tensile strength compared to the compression-moulded one. Interestingly, the extruded starch with choline chloride:urea as a plasticizer could be remoulded. 

Leroy et al. [26] used a DES based on choline chloride and glycerol or urea for plasticizing corn starch with a zein addition (10 wt%). The system was extruded and then compression moulded. After one week, the samples plasticized with a choline chloride and glycerol mixture exhibited a tendency to retrogradation (determined using XRD). In the case of the choline chloride with urea DES, such observations were noted after 12 months. 

Abbott et al. [24] applied a DES based on choline chloride with different HBDs (glycerol, urea, and ethylene glycol) for starch plasticization. New systems were compared to the starch plasticized with reline. Using glyceline (a mixture of choline chloride and glycerol in a molar ratio of 1:2) resulted in decreased flexibility and brittleness; on the other side, using ethaline (a mixture of choline chloride and ethylene glycol in a molar ratio of 1:2) resulted in a ductility increase and tensile strength reduction. Additionally, the influence of conditioning, water content, as well as processing parameters (via extrusion and thermocompression) were studied. 

Zdanowicz et al. [27] studied the influence of various plasticization methods on the properties of potato starch materials. As the plasticizing systems, the mixtures of sugars (glucose, fructose, and glycerol) were used. Starch plasticized by the components added separately exhibited a lower tensile strength and Young’s modulus than the samples plasticized with the sugar:glycerol mixture. The most beneficial effect was observed for the samples plasticized with DESs, not when separate DES components were used.

Martins et al. [28] used natural deep eutectic solvents based on glucose, sucrose, xylose, citric acid, tartaric acid, and choline chloride as plasticizing agents to enhance the supercritical foaming of the starch/poly-ε-caprolactone blend (SPCL). SPCL was plasticized by compression moulding and subsequently foamed in the presence of supercritical carbon dioxide. The porous materials were obtained, with a potential application for tissue engineering and drug delivery systems.

Zdanowicz et al. [19] compared the influence of imidazole-based deep eutectic solvents (with choline chloride, glycerol, and citric and malic acid) on potato starch and high amylose starch (Hylon II). The compression-moulded starch films containing an imidazole-based DES (choline chloride or glycerol) exhibited a reduced tendency to retrogradation. Moreover, it was concluded that the DES containing carboxylic acids could induce starch degradation.

In other work [29], the influence of binary and ternary deep eutectic solvents based on choline dihydrogencitrate on native potato starch as well as hydroxypropylated oxidized starch (HOPS) was studied. Using such a DES system resulted in the crosslinking of polysaccharide chains. Moreover, it was shown that separately added components of the DES resulted in slightly better mechanical (lower tensile strength and higher elongation at break) and barrier properties. The probable explanation could be that introducing the components gave more possibilities for an interaction with polymer chains.

In [30], using choline salts with α-hydroxylate anions (choline dihydrogencitarate, choline malate, choline acetate, and choline lactate) and glycerol as potato starch plasticizing agents has been described. Thermoplastic starch was prepared by thermocompression. Interestingly, the potato starch plasticized with choline dihydrogencitarate did not retrograde even after 12 months of storage. 

The sugar alcohol-based DES for starch plasticization applying compression moulding and extrusion techniques was reported in [31]. Additionally, the influence of premixture conditioning on thermoplastic starch properties was studied. The pre-treatment step (storage time or/and additional heating) beneficially affected the starch plasticization, e.g., the elongation at break increased slightly when compared to TPS without conditioning.

Zdanowicz [21] compared thermoplastic starches plasticized with deep eutectic solvents (based on choline derivatives and betaine) and common starch plasticizer, i.e., glycerol. The amount of dissolved content, crystallinity, and mechanical properties of prepared samples were tested. The samples containing plasticizers exhibiting the ability to dissolve starch showed a reduced degree of crystallinity and tensile strength as well as increased elongation at break when compared to those containing DESs with a limited starch dissolving ability. In another work [22], the systems based on urea, polyols, and sugars were studied. The relation between the DESs’ ability to dissolve starch and the mechanical properties of polysaccharide films (prepared via compression moulding) was observed as well. Introducing the mixtures of glycerol and urea into starch material resulted in a higher elongation at break and decreased tensile strength as well as a lowered Young’s modulus in comparison with the samples containing a DES based on glycerol, urea, and a third component. The system containing the mixture of urea with sorbitol presented a significantly lowered elongation at break similar to the samples containing sugars; in the case of using polyols, the possibility of forming hydrogen bonds between hydroxyls from polyol and polymer OH was higher, and in consequence, the mobility of the macromolecules was restricted. 

In Table 2, the DES systems used for starch plasticization were collected.

The preparation of the composites based on thermoplastic starch plasticized with a DES and with natural fillers addition was reported. Favero et al. [32] studied the influence of the zein protein addition on a thermoplastic starch plasticized by reline and glyceline. Introducing zein resulted in a tensile strength reduction when compared to neat samples. Thus, the incompatibility between zein and thermoplastic starch plasticized with a DES was suggested. 

Abbott et al. [33] prepared fibreboards from starch plasticized with a mixture of choline chloride and urea, glycerol or ethylene glycol, and with a wood flour filler. Such systems could be a replacement for urea–formaldehyde ones. Wood fibres were also applied to obtain composites (using thermocompression) from starch plasticized with a DES based on choline chloride with glycerol or urea and glycerol with imidazole [34]. It was shown that a glycerol imidazole mixture modified the surface of the wood fibres and exhibited better adhesion than the other plasticizers presented in this work.

Zdanowicz and Johannson [35] used potato starch plasticized with two or three component DESs to obtain composites with various natural fillers: sodium and calcium montmorillonite, nitrocellulose and tannin. Starch-based films were prepared via the casting method. Montmorillonite was used also to prepare (using extrusion and then compression moulding techniques) starch composites with a DES based on choline chloride and the following different HBDs: urea and imidazole [36]. Surprisingly, for the systems containing choline chloride and urea, the higher values of mechanical properties, i.e., Young’s modulus, and tensile strength were noted for composites with the lowest filler content. In another paper referring to the use of choline chloride and imidazole, such a phenomenon was not observed. 

## 4. Other DES Applications for Starch Treatment

Although the use of DESs for starch dissolution and plasticization was often reported, other applications of deep eutectic solvents could be found, i.e., as a reaction medium, copolymerization, homogenization agent, and others. 

Chen et al. [37] described the method of obtaining conductive hydrogels from starch and poly(ionic liquid). A DES consisting of acrylic acid, acrylamide, and choline chloride was successfully used in frontal polymerization with corn starch. With an increased starch content in a prepared polymer, the increase in tensile strength and conductivity was determined. Ramesh et al. [38,39] obtained polymer electrolytes from corn starch, a DES (choline chloride:urea, 1:2), and lithium bis(trifluoromethanesulfonyl)imide. The highest conductivity was noted for starch containing 80 wt% DES. The conductivity of the materials was related to the presence of the polysaccharide amorphous phase. Other researchers [40] described the method of obtaining a quasi-solid electrolyte from potato starch and a ternary DES based on choline chloride, glycerol, and urea in a different molar ratio (in comparison to the binary DES tested). In the first step, starch was chemically modified (phthaloylation) and then infused with a DES in a weight ratio of 1:3. The optimum conductivity value exhibited polyelectrolyte containing choline chloride, urea, and glycerol in a molar ratio of 4:6:2.

Deng et al. [41] used a deep eutectic solvent as a reaction medium for enzymatic starch esterification (starch laurate, starch palmitate, and starch decanoate were prepared, respectively). Akman et al. [42] used the DES system based on sulfamic acid (SAA):urea (U) (1:1) for the sulphation of potato starch. In another work [43], the optimization of the molar ratio of the DES components, the starch:DES molar ratio, and the reaction time of starch sulphation were evaluated. The optimal process conditions were determined: molar ratio of SAA:U, ca. 1:2; starch:sulphating complex, 1:3.5–1:4; and ca. 120 min, respectively.

Abbott et al. [44] used the DES to homogenize a polyethylene/starch blend. The first step was a modification of PE with a DES based on choline chloride with the following different HBDs: urea, glycerol, and ethylene glycol. The second step was extruding thermoplastic starch (plasticized with glyceline) with modified HDPE. The elongation at break of the starch:PE system increased compared to the starch and HDPE samples. Gupta et al. [45] studied the properties of high amylose starch dispersion in natural deep eutectic solvents (lactic acid:glucose—5:1, lactic acid fructose—5:1, and citric acid glycerol—1:2). The modification of the thermal and rheological properties by DES usage was confirmed.

In Table 3, the deep eutectic solvents used for starch treatment are presented.

## 5. Conclusions

Although the first reports on deep eutectic solvents are from the beginning of the 21st century, the dynamic development of this class of green solvents has been recently observed, also for starch treatment. They are studied as the potential solvents and reaction media, as well as plasticizers for biopolymers. Thus, it could be possible to obtain new, non-toxic, biodegradable materials from renewable resources, which could be a more environmentally friendly alternative for some of the currently used thermoplastic materials. The most popular systems are based on choline derivatives with glycerol or urea, but also on choline chloride with some polyols (e.g., ethylene glycol, sorbitol), and carboxylic acids (e.g., citric acid, succinic acid). The obtained starch materials exhibited various properties depending on the nature of the used plasticizer. Some of them did not show the thermoplastic starch drawbacks, such as, e.g., fast retrogradation. The interaction between polysaccharide chains and the DES components is not completely understood and deep research is required. However, the enormous number of possible combinations of components potentially allows us to give the materials tailored properties, which is a promising perspective for future development.

## Figures and Tables

**Table 2:** Deep eutectic solvents used as starch plasticizers.

Starch Origin	DES System		References
	Components	Molar Ratio	
Corn	Choline chloride: Ethylene glycol	1:2	[24]
	Choline chloride: Glycerol	1:2	[24,30,32]
	Choline chloride: Urea	1:2	[24,25,32]
Potato	Betaine: Glycerol	1:2, 1:3	[21]
	Choline acetate: Urea	1:2	[21]
	Choline bitartrate: Glycerol	1:12, 1:10, 1:8, 1:6, 1:4	[30]
	Choline chloride: Citric Acid	2:1	[16]
	Choline chloride: Glycerol	1:2	[21,27,29]
		1:3	[21]
	Choline chloride: Glycerol: Urea	1:1:1	[21]
		1:2:2	[29]
	Choline chloride: Imidazole	3:7, 2:3	[19]
	Choline chloride: Maltitol	1:4	[31]
	Choline chloride: Sorbitol	1:2	[29,31]
	Choline chloride: Sorbitol: Urea	1:1:1	[29]
	Choline chloride: Succinic acid	1:1	[16]
	Choline chloride: Urea	1:2	[16,19,21,29]
	Choline chloride: Xylitol	1:2	[31]
	Choline dihydrogencitrate: Glycerol	1:2, 1:4, 1:6, 1:8	[29,30]
		1:10, 1:12	[30]
	Choline dihydrogencitrate: Urea	1:2	[29]
	Choline dihydrogencitrate: Urea: Glycerol	1:2:2	[29]
	Choline lactate: Glycerol	1:8	[30]
	Choline lactate: Urea	1:2	[21]
	Choline malate: Glycerol	1:8	[30]
	Citric acid: Glycerol	1:8	[30]
	Citric acid: Imidazole	3:7	[19]
	Fructose: Glycerol	1:2, 1:3, 1:4, 1:5, 1:6	[27]
	Glucose: Glycerol	1:3, 1:4, 1:5, 1:6	[27]
	Glycerol: Imidazole	1:1, 3:7	[19]
	Malic acid: Imidazole	3:7	[19]
	Maltitol: Glycerol	1:6	[31]
	Sorbitol: Betaine	2:1	[31]
	Sorbitol: Glycerol	2:1, 1:2	[31]
	Sucrose: Glycerol	1:6	[27]
	Urea: Fructose: Glycerol	1:1:2	[22]
	Urea: Glucose: Glycerol	1:1:2	[22]
	Urea: Glycerol	1:1, 1:2	[22]
	Urea: Sorbitol	1:1	[22]
	Urea: Sorbitol: Glycerol	2:1:1	[22]
	Xylitol: Glycerol	1:2	[31]
Hylon VII (high amylose starch)	Choline chloride: Imidazole	3:7	[19]
	Glycerol: Imidazole	3:7, 1:1	[19]
HOPS (hydroxypropylated and oxidized potato starch)	Choline chloride: Glycerol	1:2	[29,33]
	Choline chloride: Glycerol: Urea	1:2:2	[29]
	Choline chloride: Sorbitol	1:2	[29]
	Choline chloride: Sorbitol: Urea	1:1:1	[29]
	Choline chloride: Urea	1:2	[29]
	Choline dihydrogencitrate: Glycerol	1:8, 1:6, 1:4, 1:2	[29]
	Choline dihydrogencitrate: Urea	1:2	[29]
	Choline dihydrogencitrate: Urea: Glycerol	1:2:2	[29,33]
SPCL (blend of starch and poly-ε-caprolactone)	Choline chloride: Citric Acid	1:1	[28]
	Choline chloride: Sucrose	1:1, 4:1	[28]
	Choline chloride: Xylitol	2:1, 3:1	[28]
	Citric acid: Sucrose	1:1	[28]
	Glucose: Citric acid	1:1	[28]
	Tartaric acid: Glucose	1:1	[28]

**Table 3:** Deep eutectic solvents for starch treatment.

Application	Starch Origin	DES System		References
		Components	Molar Ratio	
chemical modification	Corn	Acrylic acid: acrylamide: Choline chloride	1:1:1	[37]
	Potato	Sulfamic acid: Urea	1:1, 1:2, 1:3	[43]
polymer electrolyte preparation	Corn	Choline chloride: Urea	1:2	[38,39]
	Potato	Choline chloride: Glycerol	1:2	[40]
		Choline chloride: Urea	1:2	[40]
		Choline chloride: Urea: Glycerol	4:7:1, 4:6:2, 4:5:3, 4:4:4, 4:3:5, 4:2:6, 4:1:7	[40]
homogenisation	Corn	Choline chloride: Ethylene glycol	1:2	[44]
		Choline chloride: Glycerol	1:2	[44]
		Choline chloride: Urea	1:2	[44]
reaction medium	n.d. *	Choline chloride: Ethylene glycol	2:1	[41]
starch dispersion	Joymoti rice	Citric acid: Glycerol	1:2	[45]
		Lactic acid: Fructose	5:1	[45]
		Lactic acid: Glucose	5:1	[45]

* no data.

## Data Availability

Not applicable.
